# A Green HPLC Approach to Florfenicol Analysis in Pig Urine

**DOI:** 10.3390/ph17040495

**Published:** 2024-04-12

**Authors:** Ksenija Šandor, Eleonora Perak Junaković, Svjetlana Terzić, Irena Žarković, Anja Vujnović, Dominika Fajdić, Mirta Pehnec, Sonja Sinković, Irena Ćaleta, Miroslav Andrišić

**Affiliations:** 1 Laboratory for Analysis of Veterinary Medicinal Products, Croatian Veterinary Institute, Savska Cesta 143, 10000 Zagreb, Croatia; sandor@veinst.hr (K.Š.); terzic.svjetlana@gmail.com (S.T.); zarkovic@veinst.hr (I.Ž.); vujnovic@veinst.hr (A.V.); fajdic@veinst.hr (D.F.); pehnec@veinst.hr (M.P.); sinkovic@veinst.hr (S.S.); andrisic@veinst.hr (M.A.); 2 Chemistry, Selvita Ltd., Prilaz Baruna Filipovića 29, 10000 Zagreb, Croatia; irena.caleta@selvita.com

**Keywords:** florfenicol, HPLC-DAD, greenness assessment, swine, urinary excretion

## Abstract

Florfenicol (FF) is a broad-spectrum antibiotic used to treat gastrointestinal and respiratory infections in domestic animals. Considering FF’s rapid elimination via urine after drug treatment, its use increases concerns about environmental contamination. The objective of the study was to establish a sustainable chromatographic method for simple analysis of FF in pig urine to investigate the urinary excretion of FF after a single intramuscular administration of 20 mg FF/kg body weight. The urine sample was prepared using a centrifuge and regenerated cellulose filter, and the diluted sample was analyzed. The method was validated in terms of linearity, the limit of detection (0.005 µg/mL) and quantitation (0.016 µg/mL), repeatability and matrix effect (%RSD ranged up to 2.5), accuracy (varied between 98% and 102%), and stability. The concentration-time profile of pig urine samples collected within 48 h post-drug administration showed that 63% of FF’s dose was excreted. The developed method and previously published methods used to qualify FF in the urine of animal origin were evaluated by the National Environmental Method Index (NEMI), Green Analytical Procedure Index (GAPI) and Analytical GREENness Metric Approach (AGREE). The greenness profiles of published methods revealed problems with high solvents and energy consumption, while the established method was shown to be more environmentally friendly.

## 1. Introduction

**Figure 1:**
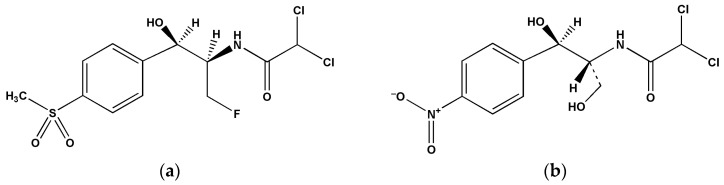
The chemical structure of: (**a**) florfenicol; (**b**) chloramphenicol.

Florfenicol (FF) is a known synthetic amphenicol widely used in veterinary medicine as a broad-spectrum antibiotic (Figure 1a). It is effective against most Gram-positive and Gram-negative bacteria isolated from domestic animals and commonly used for the treatment of diseases related to gastrointestinal and respiratory tract infections [1,2,3,4]. It is characterized by high bioavailability, good tissue penetration and, in general, regardless of the route of administration, rapid elimination. Its primary mechanism of elimination is urinary excretion in a biologically active form, and studies conducted on pigs have shown that about 70–80% of intramuscularly or orally administered FF doses are excreted within two weeks [5]. Recent monitoring studies of antibiotic use in animal breeding have affirmed a high excretion rate of active substances in feces and urine in their non-metabolized form [6,7], confirming that this also contributes to environmental contamination with antibiotics [8,9]. Those studies are usually focused on contaminated matrices such as water, soil or food since manure and slurry, as natural fertilizers widely used in agriculture, represent the most common mediums for spreading antibacterial residues in nature. However, due to sampling simplicity, urine samples have lately showed to be a helpful control matrix for surveillance purposes as one of the informative sources for acknowledgement of the magnitude of contamination with specified antibiotics in animal farms [10]. It can also be an alternative sample to edible tissues, for example, for monitoring antibiotic administration [11] or detecting an appropriate time of animal slaughter [12].

Analytical methods for determination of the FF and/or its metabolite florfenicol amine (FFA) in blood samples, edible tissues or drug formulations are primarily described in the literature. For example, the following methods were used: voltammetric method [13], enzyme-linked immunosorbent assay (ELISA) method [14], ultraviolet-visible (UV-Vis) spectrophotometric method [15], near-infrared spectroscopy [16], capillary electrophoresis [17], and chromatographic methods such as thin-layer chromatography (TLC) [18], gas chromatography (GC) [19], (ultra)high-performance liquid chromatography ((U)HPLC) with ultraviolet (UV), diode array detector (DAD) or fluorescence (FL) detector [20,21,22,23,24,25], and liquid chromatography-tandem mass spectrometry (LC-MS/MS) [26,27,28,29]. Only several reported papers included and described the analytical methods for determining the FF in urine samples of animal origin [1,11,12,30,31,32,33]. However, employing diverse and often complex analytical techniques in FF analyses indicated that their impact on the environment should also be considered. Since the 2000s, from an environmental perspective, there has been a trend in analytical chemistry towards greening reversed-phase (RP) HPLC methods. Green Analytical Chemistry (GAC), as a part of Green Chemistry, aims to eliminate or reduce the use of hazardous substances in analytical methods. Developing methods according to the 12 principles of GAC makes analytical procedures less environmentally harmful and more human-friendly regarding less energy consumption, cost reduction and increased operator safety [34,35]. The National Environmental Method Index (NEMI), Analytical Eco-Scale (AES), Green Analytical Procedure Index (GAPI) and Analytical GREENness Metric Approach (AGREE) are well-known qualitative and quantitative greenness metrics tools, very helpful in greenness evaluation of the analytical method [34,36,37,38]. Most metric tools are accessible as freeware bases or software, making the greenness calculations more straightforward. Even though the available developed metrics are continuously improved, and new ones are being created, the above mentioned tools are sufficient to provide reliable and factual results in assessing the green aspects of the analytical method. 

The main objective of this study was to establish a RP-HPLC-DAD method for simple analysis of FF in pig urine and to evaluate its greenness simultaneously by NEMI, AES, GAPI and AGREE metric tools. From GAC’s point of view, the proposed method’s development was focused on the miniaturization of the sample preparation procedures and reducing the volume and toxicity of solvents, energy consumption and waste. Finally, the established method was employed in the urinary excretion study of FF on urine samples collected from crossbred fattening pigs intramuscularly treated with a single dose of 20 mg FF/kg body weight (bw). This article also provides a mini-review of FF determination methodologies in urine samples of animal origin. The greenness aspects of the established RP-HPLC-DAD method and previously published methods were compared, and their differences were summarized.

## 2. Results

### 2.1. RP-HPLC-DAD Method Validation

In our study, the parameters related to estimating the well-known performance quality characteristics of the bioanalytical methods were considered, and all validated parameters met the requirements. Validation results are depicted in Table 1, and additional data are presented in the Supplementary Files (Table S1 and Figure S1).

The specificity of RP-HPLC-DAD method was confirmed by determining the retention times of the chromatographic peaks, i.e., FF and internal standard chloramphenicol (CAP, Figure 1b), and their UV spectra. The chromatograms obtained from the blank urine samples of pigs from the control group and blank urine samples spiked with FF and CAP were also compared. Resolution data (*R_s_* > 3) provided by system suitability tests (Table S1) and the chromatograms (Figure S1) showed good separation between FF and CAP analytes and the other urine impurities, ensuring a good selectivity of the developed method.

Assessment of the potential matrix effect was carried out due to its importance for the method’s applicability. Results of the sample preparation repeatability (%RSD) for the blank urine samples spiked before (pre-extraction spiked urine) and after (post-extraction spiked urine) sample treatment protocol with the FF and CAP at low and high concentrations ranged from 1% to 2.5% (Table 1). Recoveries varied from 98% to 102%. Finally, the matrix effect (*ME%*) in post-extraction spiked urine was quantitatively evaluated for FF and CAP using Equation (2). Results were acceptable for both analytes. No significant differences were observed between reference standard solutions and spiked sample solutions (one-way analysis of variance, ANOVA, *p* > 0.05), demonstrating that the matrix did not influence the extraction of analytes. Moreover, the carry-over effect was studied, and no ghost peaks or effects on the response of FF or CAP were observed. This indicates that the applied washing procedure between sample injections into the column of the HPLC instrument was sufficient.

In linearity studies, calibration curves of reference standard solutions and urine samples spiked with the FF in the concentration ranges of 0.05–20.0 μg/mL were established. The concentrations of the FF analyte were calculated from calibration curves using the internal standard method and validated by the linear regression model. The data summarized in Table 1 confirm that the proposed RP-HPLC-DAD method provides good linearity (*p* < 0.05) for urine samples with a correlation coefficient greater than 0.995. The limit of detection (LOD) and the limit of quantification (LOQ) of FF in urine were 0.005 and 0.016 μg/mL, respectively. The accuracy of the method was demonstrated by recoveries, which vary between 98% and 102%, while repeatability was up to 2% RSD. Stability studies of urine samples spiked with FF and CAP standards showed that significant changes were caused by storage at a temperature of 5 ± 3 °C (ANOVA, *p* < 0.05) after three freeze-thaw cycles within seven days. A decline of FF level was up to 11%, demonstrating that it is necessary to freeze the samples at −20 ± 5 °C after assays and re-assays.

### 2.2. Green Profile of the RP-HPLC-DAD Method

The results obtained using the NEMI, AES, GAPI, and AGREE green tools in the evaluation of the RP-HPLC-DAD method established for FF determination in pig urine are summarized in Table 2. 

The hazardous quadrant in the NEMI pictogram indicates that toxic and highly flammable chemicals were utilized, methanol as a solvent for stock standard solutions and acetonitrile as component B of the mobile phase. The mobile phase did not pose a corrosive threat to the environment because the pH was 3.5, and no bioaccumulative and toxic chemicals (PBT) were used. The produced waste was less than 50 g. 

The method achieved an AES rank of 75 scores, where the highest penalty points were assigned to the waste (6 points) and previously mentioned highly flammable chemicals (4 and 3 points). The collection (no. 1), preservation (no. 4), used solvents/reagents (no. 7) and waste (no. 14) were indicated with red pictograms in the GAPI assessment as non-eco-friendly steps in our method. 

Even though the overall AGREE score of our method is 0.54 on a maximum scale of 1.00, the representation in the middle of the clock-like graph is light green colored. The AGREE results also indicate the phases with environmental impact previously determined by NEMI, AES and GAPI. However, due to a more rigid evaluation considering the aspects overlooked in the AES and GAPI assessments, AGREE revealed a new impact on the environmental sustainability of the RP-HPLC-DAD method. It is attributed to the sample throughput (principle no. 8), i.e., only two consecutive samples can be analyzed in one hour.

### 2.3. Greenness Assessments of the Published Methods

The articles describing methods for FF determination in urine samples of animal origin were collected from Medline, PubMed, SciFinder, ScienceDirect, Scopus and Web of Science databases from 1981 to January 2024. The literature review revealed that previously published methods had not been evaluated by any environmental assessment tool. Therefore, to our knowledge, the results depicted in Table 3 are their first reviews covered by the greenness assessment.

The environmental aspects of the established RP-HPLC-DAD method were compared with published analytical methods. Accordingly, the comparative assessments provided by NEMI, GAPI, and AGREE tools demonstrate better environmental performances of the established method. The RP-HPLC-DAD method has more green (three) and yellow (seven) and less red (four) GAPI zones with the highest AGREE score of 0.54. Furthermore, a comprehensive evaluation revealed that among published methods, only the method reported by Azzouz and Ballesteros [30] is close to the AGREE results of our method. However, their method has no data on waste treatments, more than four red GAPI zones, including the type of quantitative method, and it cannot be considered an ecological method.

### 2.4. Urinary Excretion of the FF

**Figure 2:**
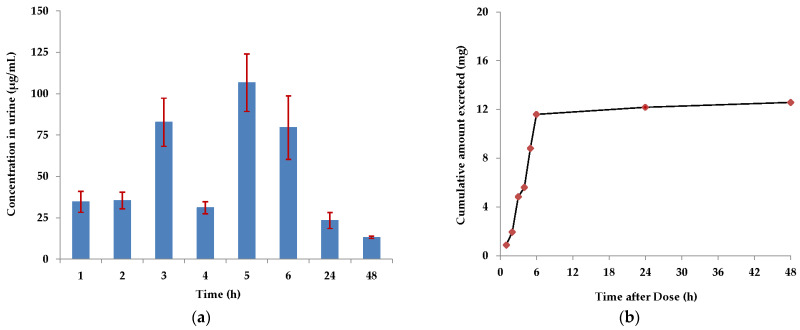
Single intramuscular administration of 20 mg florfenicol/kg body weight in pigs: (**a**) Mean concentration of florfenicol in urine ± standard error of the mean, *n* = 6; (**b**) Cumulative amount of veterinary medicinal product excreted in the urine.

The FF concentration-time profile of urine samples collected at selected intervals between 1 to 48 h after a single intramuscular (IM) administration of veterinary medicinal product (VMP) to pigs is presented in Figure 2a. 

The obtained profile revealed two peaks of FF with concentrations of 82.76 ± 14.54 µg/mL and 106.70 ± 17.37 µg/mL at the third and the fifth-hour post-administration, respectively. However, a specific decrease in the FF concentration was observed in urine samples between 3 h (82.76 ± 14.54 μg/mL) and 4 h (31.14 ± 3.68 μg/mL), followed by a significant increase at 5 h post-drug administration. After that, the levels of FF in urine samples were continuously decreased, and at 48 h, the concentration of 13.24 ± 0.74 µg/mL was quantified.

Urinary excretion rates were determined using Equation (1). The cumulative excreted amounts of FF at various time points, illustrated in Figure 2b, were calculated as described in Section 4.3. The calculated maximum urinary excretion rate of 1.73 mg/h was achieved at intervals from zero to one hour, confirming that FF is rapidly excreted via urine after VMP administration. The cumulative amount of FF excreted in urine by 48 h was 12.57 ± 0.02 mg, and the percentage of the administered dose recovered in urine was 62.85%. Figure S1d, presented in the Supplementary File, displays a representative chromatogram of the urine sample of the treated pigs.

## 3. Discussion

A green approach in analytical methodology, development, optimization, and validation of critical analytical parameters (specificity, selectivity, LOD, LOQ) is a significant challenge but nowadays highly recommended. Still, the survey of published analytical methods for FF analysis in urine samples of animal origin did not disclose any data on their green profiles. Moreover, the complexity of the methodologies and the lack of information on the sample (collection, transport) and waste treatments were observed. Therefore, in this research, the RP-HPLC-DAD method for FF analysis in pig urine samples following the principles of GAC was established, and a critical review of its final green profile was given.

As a highly lipophilic compound, FF (Figure 1a) required the RP-HPLC as the method of choice. Furthermore, since the sample was diluted urine, the internal standard method was used to ensure the accuracy and precision of quantitation as well as the robustness of the method. Adding the internal standard at an early stage of sample preparation can elucidate and correct variations or losses caused by sample preparation, matrix effects, chromatographic separation or detection. Generally, internal standards are commonly used in chromatographic bioanalysis. However, the selected internal standards should have as similar physicochemical properties as possible to the analytes of interest. Therefore, the CAP (Figure 1b), a first-in-class amphenicol, was chosen as an internal standard. Moreover, it is an amphenicol that should not expected to be found in pig urine samples since it has been banned in food-producing animals for over 25 years. The conditions of the HPLC-DAD method described in our previous experimental study of FF and FFA in pig cerebrospinal fluid [24] were chosen to achieve good peak symmetry and resolution of FF and CAP in urine analysis. 

In order to implement the GAC concept, the goal was to include the reduction of solvents and energy consumption as a greening criterion. It was accomplished by reducing steps in sample preparation without using the standard extraction procedures, only employing centrifugation and filtration as pre-treatment of the urine for direct analysis. Furthermore, equipment and instrumentation with an energy consumption of less than 1.5 kWh/sample were used (Table 2). The gradient mode of elution was justified as it presented better chromatographic resolution than isocratic. The optimization of the proposed method was characterized by a mobile phase with a volume fraction of acetonitrile up to 4% per run, which is a significant reduction of acetonitrile compared to similar methods [1,31]. Generally, acetonitrile is an organic solvent of choice in FF chromatographic analyses, but it can be substituted with methanol [11]. Replacement of the acetonitrile with a bio-based solvent is always recommended from an environmental perspective due to its toxicity and environmental life cycle impacts from production to waste recycling [39,40,41]. Still, the replacement can raise problems related to high system back pressure in water mixtures and higher UV absorbance cut-off wavelength, resulting in the deterioration of detection sensitivity. In the recent pharmacokinetic study of FF in donkey urine [33], the chromatographic method reported by Somogyi et al. [29] was applied using a mobile phase that contained only 5% acetonitrile. However, Liu et al. [33] used significant quantities of acetonitrile (>20 mL/sample) in donkey urine sample preparation. This demonstrates that the choice of solvents is often a compromise governed by many factors like technical, physicochemical or sustainability criteria, as well as the new knowledge about green recovery processes of hazardous solvents [42]. 

Urine sample represents a matrix with a low-cost and less invasive collection method than blood or tissue sample collection. Some studies have already shown the successful use of urine as a control matrix by finding a higher frequency and concentrations of antibiotics in urine than in muscle tissue samples [11]. The defined characteristics of the RP-HPLC-DAD method enable good response of individual analytes, adequate separation efficiency of FF and CAP, high symmetry of the peaks and shortening of the analysis time (Table S1 in the Supplementary File). The approach without sample preparation beyond dilution and filtration demonstrates that the analysis of urine samples can be simplified. Validation data confirms the method’s selectivity, sensitivity, accuracy and precision (Table 1), which was demonstrated successfully on urine samples from treated pigs (Figure S1 in the Supplementary file). Therefore, we believe that this analytical procedure could also be a helpful initial tool in determining or monitoring FF-level exposure on farms.

One of the objectives of this study was to demonstrate the method’s sustainability and evaluate its greenness with the help of qualitative and quantitative metrics tools. Therefore, the RP-HPLC-DAD method was studied using NEMI, AES, GAPI, and AGREE greenness assessment tools (Table 2). The simultaneous application of these tools enabled the evaluation of the method’s green characteristics and its weakest points. Although the NEMI is one of the initial standards for the green profile evaluation, its pictogram simply displays a “green or white” quadrant of the analytical procedure. It does not have a scale to provide details if the value of the method is below or above a certain threshold. Therefore, the white quadrant in the NEMI pictogram of the RP-HPLC-DAD method only qualitatively indicates the use of reagents with specific health and safety hazards. As a semi-quantitative tool, the AES represents a tool that can provide evaluation to compare different analytical methods. Thus, the AES penalty result demonstrates a minimal negative impact of the RP-HPLC-DAD method on human health and the environment considering occupational hazards, the energy consumption of used instruments and waste. Even though the AES tool provides more information about different aspects of our procedure than NEMI, it is necessary to emphasize its disadvantages related to the lack of additional quantifiers of analytical procedure for example, the discrimination between micro and macro scales. Given that the AES tool makes it difficult to specify all critical aspects of the analytical procedure, further assessments were carried out using its upgraded versions, the GAPI and AGREE tools. These quantitative profiles facilitate visualization and indicate where and how to intervene in the critical phases of the RP-HPLC-DAD method. The GAPI and AGREE evaluation highlighted the advantages of the established method, particularly the absence of extraction and derivatization phases. However, the coherence of the sample collection and device positioning (off-line analysis), solvent toxicity and amount of waste are weak points affirmed in the RP-HPLC-DAD method’s greening.

As mentioned in the Introduction section, green tools are constantly being improved to meet the needs of the chemical community. The Complementary Green Analytical Procedure Index (ComplexGAPI) and Analytical GREENness Metric for sample preparation (AGREEprep) are new specific tools that represent significant steps forward in the gold standardization of green method profile evaluation [43,44]. AGREEprep is a green tool specifically designed to evaluate the sample preparation phase in the analytical procedure. It is a tool that provides good accuracy in detecting and predicting the aspects that can be improved in sample preparation. Furthermore, ComplexGAPI is a tool developed not only to upgrade the GAPI tool but also to provide factual results of additional fields that were performed before the analytical procedure. However, in our study, the AGREEprep and ComplexGAPI were not used to assess the greenness of the established RP-HPLC-DAD method, as the simplified approach was considered for its comparison with similar published methods. Moreover, the deficiency of data regarding pre-analyses processes of the established method (for example, synthesis and manufacturing of organic compounds/regents and stationary phase), as well as the lack of some required conditions and/or quantifiers in the published methods were perceived.

In this paper, a step forward has been made for published methods used for FF analyses in the urine of animal origin since its greenness assessments have not been published. Their greenness was assessed using NEMI, GAPI and AGREE tools (Table 3). The methodology used in the study recently reported by Liu et al. [33] was not assessed due to insufficient data on chromatographic conditions (column dimensions, elution mode, etc.). The GAPI assessment of the published methods revealed that liquid-liquid extraction (LLE) or solid-phase extraction (SPE) procedures and the most energy-consuming instrumental techniques (>2.5 kWh/sample), such as GC/MS [30] or LC-MS/MS [11,32] were used for FF analyses. Although the method reported by Li et al. [32] utilizes only centrifugation in urine sample preparation, the AGREE assessment resulted in an overall score of 0.4. It is due to the complexity of the indirect competitive ELISA method, manifested in the synthesis of haptens, purification of antigens/antibodies and precipitation procedures. As can be perceived in all GAPI profiles of the methods displayed in Table 3, the deficiency of data concerning the collection, preservation, transport and storage of the urine samples before preparation for analysis or waste treatments was present. Nevertheless, the exacting of their procedures was still reflected on AGREE profiles, where overall scorers were below or equal to 0.50. 

The greenness assessment results facilitate insight into additional improvements for better sustainability of the proposed method. From an analytical perspective, Pastor-Belda et al. [45] took a step further in human urine sample preparation for amphenicols analyses using a new-generation technique of miniaturized extraction procedures, a dispersive liquid-liquid microextraction (DLLME). Accordingly, we should also consider the DLLME approach and UPLC instrumental technique with a DAD or FL detector in further steps of the method optimization. From a GAC perspective, Manousi et al. [46] recently introduced a new green tool, a Blue Applicability Grade Index (BAGI), complementary to the GAPI, ComplexGAPI, AGREE and AGREEprep tools. Thus, in the following analytical studies, it is necessary to consider the application of the BAGI tool to present and compare the practicality and applicability of the RP-HPLC-DAD method with newly “green-optimized” methods for analyses of FF in pig urine.

Despite there have been published various pharmacokinetic studies of FF in pigs, to our knowledge, the urinary excretion study of FF after a single IM administration of 20 mg FF/kg bw has not been described in the literature. The rapid and high excretion rates of FF within 48 h after drug administration were established in this urinary study using the proposed RP-HPLC-DAD method (Figure 2). FF was detected in its unchanged form in all pig urine samples. The results also demonstrate that peak concentrations of 100 µg FF/mL can be expected in urine 5 h after drug administration and that more than 10 µg/mL of FF can be quantified 48 h after its administration. Even though, the experimental study on pigs, sample storage, preparation and analyses were carried out under the same conditions and protocols for all experimental animals, a decrease in the concentration of FF in urine samples between 3 h and 4 h post-drug administration was significant. This phenomenon is probably due to the drug’s pharmacokinetics, as all animals showed decreased drug concentration during the specified period. Overall, the obtained data acknowledge the fact that up to 70% of the FF dose after IM drug application is excreted via urine in its non-metabolized form [5], which is an amount that can significantly contribute to environmental antibiotics contamination.

Shedding of the drug via urine and its persistence in the environment are some of the factors that make control of antibiotic contamination and antimicrobial resistance difficult. The results in this research additionally confirm the necessity of systematic surveillance of the FF release to the farm’s surroundings. Moreover, we consider it essential since recent findings revealed an association between human exposure to FF and increased risk to human health, particularly in children [47,48,49,50].

## 4. Materials and Methods

### 4.1. Sample Collection

Urine samples were taken from untreated pigs that served as a control group and pigs treated with the VMP during the animal experiment described in the pig cerebrospinal fluid experimental FF’s study [24]. The experimental research on crossbred fattening pigs was approved by the Ethics Committee of the Faculty of Veterinary Medicine, the University of Zagreb, Croatia (reference 640-01/06-17/30, File no. 61-01/139-06-80). Briefly, pigs with an average weight of 80 kg were housed on a commercial farm, and they were fed with standard commercial feed and had water *ad libitum*. Treated pigs were subjected to an FF treatment with Nuflor Injectable Solution 300 mg/mL (MSD/Schering-Plough A.H., Luzern, Switzerland). The single dosage of 20 mg FF/kg bw was given intramuscularly using a 16-gauge needle into the neck muscles. A urine sample was collected per pig in a sterilized polypropylene container cup before administration and at 1, 2, 3, 4, 5, 6, 24 and 48 h post-administration. The collected volumes of urine samples ranged from 25 to 30 mL. All urine samples were stored at −20 °C until analysis in the laboratory.

### 4.2. Standard and Sample Preparation

The analytical standard of FF (Sigma-Aldrich, Steinheim, Germany) and internal standard CAP (Dr Ehrenstorfer, Augsburg, Germany) were used. HPLC-grade acetonitrile, methanol and water were provided by Merck (Darmstadt, Germany). Phosphoric acid (85 wt% in water) of analytical grade was purchased from Kemika (Zagreb, Croatia).

The methanol stock solution of FF was diluted to 20.0, 15.0, 10.0, 7.5, 5.0, 2.5, 1.0, 0.5, 0.1 and 0.05 µg/mL by adding component A of the mobile phase for obtaining the calibration curve. The appropriate volumes for each dilution were also added to blank urine to prepare urine FF standards ranging from 0.05 to 20.0 µg/mL. The concentration of internal standard CAP in all calibrators was 10.0 µg/mL. 

Frozen urine samples were first thawed at room temperature and then centrifuged (Centric 322A, Tehtnica Železniki d.o.o., Železniki, Slovenia) at 4500 rpm for 15 min before sample treatments. The 500 µL of a urine sample was spiked with 0.1 mL of the internal standard stock solution and diluted with component A of the mobile phase. Samples were then vortexed (Assistent Reamix 2789, Karl Hecht GmbH, Fritzens, Germany) for 1 min and filtered using a regenerated cellulose filter (pore size 0.45 μm, Agilent Technologies, Waldbronn, Germany).

### 4.3. RP-HPLC-DAD Method 

Chromatographic separation was performed by the TSP SpectraSystem HPLC system (Thermo Separation Products, San Jose, CA, USA) with a DAD detector. The temperature of the reversed phase C-8 column (Zorbax SB RP C-8; 250 × 4.6 mm ID, particles: 5 μm; Agilent Technology, Santa Clara, CA, USA) was maintained at 30 °C, and the injection volume was set to 30 μL. The mobile phase consisted of component A containing water and acetonitrile (80:20 *v*/*v*; pH = 3.5 with phosphoric acid) and acetonitrile as component B. A total flow of 1.0 mL/min was used. The pH-meter (Schott Geräte GmbH, Mainz, Germany) with electrode Blue Line 14 pH (SI Analytics GmbH, Mainz, Germany) was used for the pH adjustments of the component A. The gradient HPLC method was used within 20.0 min in the following sequence: initially, mobile phase composition changed linearly from 100% to 80% (*v*/*v*) component A for 18.0 min; then continued linearly from 80% (*v*/*v*) component A and 20% component B (*v*/*v*) to 100% (*v*/*v*) component A for 0.5 min; and maintained at 100% (*v*/*v*) component A for 1.5 min. The autosampler needle was flushed with 300 μL of methanol between injections of different urine samples to remove any residuals. The absorbance was measured at a wavelength of 224 nm. Data acquisition was achieved by ChromQuest Software Version 5.0 (Thermo Fisher Scientific, San Jose, CA, USA).

The established RP-HPLC-DAD method was applied to the excretion study of the FF in urine samples obtained from pigs that received a 20 mg dose of FF/kg bw intramuscularly. The collection scheme for urine was 0–1 h, 1–2 h, 2–3 h, 3–4 h, 4–5 h, 5–6 h, 6–24 h, and 24–48 h. Observations included times of urine collection interval (*dt*), midpoint of urine collection (t*), volumes of urine collected and concentration of unchanged drug in each sample (*X_u_*). The volume of urine collected, *V* (mL), was measured directly. After the data was determined, the amount of FF excreted in time of interval (*dX_u_/dt*) was calculated and then plotted against the median of the collection interval to calculate the urinary excretion rate as presented in Equation (1). Cumulative amount of FF excreted (*X_u_^t^*) was obtained by summation after each collection interval.

*The urinary excretion rate* (mg/h) = (*dX_u_*/*dt*)/t* (1)

### 4.4. Method Validation

The RP-HPLC-DAD method was validated according to the requirements for the bioanalytical method validation [51]. The selectivity of the developed method was estimated by analyzing blank urine samples and blank urine samples supplemented with known concentrations of FF. The potential matrix effect (*ME*) was evaluated in a relevant medium by comparing the response of the analytes in standard and matrix solutions. The first medium, an analytical standard prepared by dissolving in component A of the mobile phase, was considered the analytical signal reference with no matrix effect. The second one corresponded to the fortification of the blank urine with the FF and CAP before the sample treatment protocol described in Section 4.2 (pre-extraction spiked urine). The third medium was prepared by fortifying extracted urine with the FF and CAP (post-extraction spiked urine). The percentage of matrix effect (*ME*%) in post-extraction addition protocol was calculated following Equation (2), where X was the measured value of the analyte in a standard solution, and Y was the measured value of the analyte in a urine sample spiked with the analyte at the same concentration as the standard solution after sample preparation. The conditions of the method may allow the matrix effect of up to 5%.

*ME*% = ((X − Y)/X) × 100 (2)

Linearity was evaluated by standard solutions and spiked urine calibrators at ten different concentrations of FF in the range from 0.05 to 20.0 µg/mL measured in triplicate. The correlation coefficients (*R*^2^) of the calibration curves were calculated by linear regression using the ratio of the standard area to the internal standard area against the analyte concentration. The limit of detection (LOD) and limit of quantification (LOQ) were defined by LOD = 3.3 *δ*/*S* and LOQ = 10 *δ*/*S*, respectively, where, *δ* is the standard deviation of response, and *S* is the slope of the calibration curve. The potential carry-over effect was studied through the subsequent injection of spiked urine samples at the highest calibration level of 20.0 μg/mL. The quantifications of spiked samples were performed at three concentration levels (1, 5 and 10 µg/mL) to ensure the accuracy of the proposed method. Intra-day precision (repeatability) and inter-day precision (intermediate precision) were defined by RSD% and estimated from the six-fold analysis of the same standard solution or spiked urine sample on the same day (intra-day) and three successive days (inter-day), respectively. 

The obtained data of spiked and non-spiked solutions in the stability study were statistically analyzed by the one-way ANOVA (Microsoft Excel 2016). A difference probability of *p* < 0.05 was deemed statistically significant.

### 4.5. Greenness Assessment

Apart from the analytical criteria assessed during method validation, the parameters of the developed method were also estimated considering the method’s ecological aspects. The established method’s greenness and the greenness review of the methods for determining FF in urine described in the literature were analyzed using the following tools: NEMI, AES, GAPI and AGREE. 

The NEMI pictogram is represented by a circle with four green or colorless quadrants in which the colorless quadrant presents the profile of the persistent, bioaccumulative and toxic chemicals (PBT), hazardous chemicals, corrosive (pH during the analysis is <2 or >12) and waste (>50 g) [34]. This green metric is associated with the U.S. Environmental Protection Agency (EPA), Toxic Release Inventory (TRI) and the Resource Conservation and Recovery Act (RCRA) lists [52,53].

Penalty points for AES assessment calculation are based on reagent amounts and hazards, energy consumption, occupational hazards and waste [36,54]. The AES score for the analytical method ranges from 0 to 100: >75 excellent, >50 acceptable, and <50 inadequate. 

A specific symbol with five pentagrams represents the GAPI tool. Evaluation in GAPI includes fifteen parameters that observe the greenness of the method from the sampling procedure to the final analysis. The colored field of the pictogram indicates the ecological impact on the environment: red “high”, yellow “lower” and green “safe” effects. The blank field means that there is no information for the evaluation of certain parameters. The absence of the field displays that it is not applicable. The circle in the middle of the pictogram represents the procedure for qualification and quantification [37]. 

AGREE software was downloaded from https://mostwiedzy.pl/AGREE (accessed on 1 March 2024) and used to estimate the twelve SIGNIFICANCE principles: sample treatment, sample amount, device positioning, sample preparation stages, automatization/miniaturization, derivatization, waste, analysis throughput, energy consumption, source of reagents, toxicity and operator’s safety [38].

## 5. Conclusions

In summary, to our knowledge, this is the first report on greenness assessment of the methodologies used for FF determination in urine samples of animal origin. The proposed RP-HPLC-DAD method, established for the experimental study of urine in pigs intramuscularly treated with a single dose of 20 mg FF/kg bw, proved to be more sustainable than similar methods. Furthermore, due to the simplicity of sampling, urine has shown to be a very suitable and cost-effective sample for rapid estimation of FF exposure. The results of the urinary excretion study demonstrate the rapid elimination of a major fraction of the FF dose in its unchanged form. These findings affirm concerns about environmental contamination with amphenicols and emphasize the importance of systematic surveillance of the FF release to the farm’s surroundings.

## Figures and Tables

**Table 1:** Linearity, limit of detection, limit of quantification, precision, accuracy, matrix effect and stability data for the florfenicol (FF) determination in pig urine using an internal standard method with chloramphenicol (CAP).

Parameters	Standard Solution	Urine Solution
Range (µg/mL)	0.05–20.0	0.05–20.0
Correlation coefficient, *R*^2^	0.998	0.997
Regression, *p*-value	<0.05	<0.05
Limit of detection (µg/mL)	0.001	0.005
Limit of quantification (µg/mL)	0.004	0.016
Intra-day precision (%RSD, *n* = 6)	0.48	0.32
Inter-day precision (%RSD_pooled_, *n* = 18, 3 days)	0.55	1.12
Accuracy at a nominal concentration of: *		
2%	101.32 ± 1.16	102.53 ± 2.24
100%	99.75 ± 0.20	99.41 ± 1.71
200%	100.95 ± 0.16	100.36 ± 1.73
Pre-extraction spiked urine (recovery% ± %RSD, *n* = 6):		
FF at 1.0 µg/mL		98.48 ± 1.17
CAP at 1.0 µg/mL		101.77 ± 1.84
FF at 10.0 µg/mL		101.93 ± 2.49
CAP at 10.0 µg/mL		98.78 ± 0.83
Post-extraction spiked urine (*ME*% ± SD, *n* = 6):		
FF at 1.0 µg/mL		1.03 ± 0.35
CAP at 1.0 µg/mL		1.88 ± 1.28
FF at 10.0 µg/mL		2.27 ± 0.51
CAP at 10.0 µg/mL		2.10 ± 1.96
Stability after 24 h at 5 ± 3 °C **	>0.05	>0.05
Stability after 168 h at 5 ± 3 °C **	>0.05	<0.05
Stability after 24 h at −20 ± 5 °C **	>0.05	>0.05
Stability after 168 h at −20 ± 5 °C **	>0.05	>0.05

* Nominal concentration was 5 µg FF/mL. Accuracy results were presented as mean ± relative standard deviation (%RSD), *n* = 6. ** Stability results were statistically analyzed by the one-way analysis of variance and presented as *p*-value. A *p*-value of <0.05 was considered statistically significant.

**Table 2:** Green profile of an established method for the analysis of florfenicol in pig urine samples.

Steps	NEMI	AES	GAPI	AGREE
Sample treatment			Collection (1): offline	Sample procedure (1): offline Sample amount (2): 0.5 mL Device positioning (3): off line Preparation stages (4): 3 Automatization (5): semi- automatic; miniaturized
			Preservation (2): none	
			Transport (3): required	
			Storage (4): at −20 °C	
			Type of method (5): simple	
			Scale of extraction (6): - *	
			Solvents/reagents used (7): non-green	
			Additional treatments (8): filtration	
Reagents and solvents		Chloramphenicol: 3	Amount (9): <10 mL Health hazard (10): NFPA_score_ ** 2 or 3 Safety hazard (11): NFPA_score_ ** 2 or 3	Derivatization (6): none Source of regents (10): 2 bio-based Toxicity (11): 8.2 mL
		Methanol: 3		
		Acetonitrile: 4		
		Phosphoric acid: 2		
Instruments		Transport: 1	Energy (12):	Waste (7): 42.1 mL Analysis per hour (8): 2 analytes/h Energy consumption (9): LC Operator’s safety (12): highly flammable
		Storage: 2	<0.1 kWh/sample: pH-meter	
		pH-meter: 0	≤1.5 kWh/sample:	
		Centrifuge: 1	Centrifuge	
		Vortex: 1	Vortex	
		Sonicator: 1	Sonicator	
		HPLC-DAD: 1	HPLC-DAD	
	Occupational hazard: 0		Occupational hazard (13): hermetic	
		Waste: 6	Waste (14): ≥42 mL	
		∑ total PP ***: 25	Waste treatment (15): recycling ****	
Pictogram and/or score	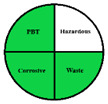	75	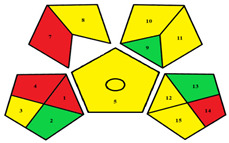	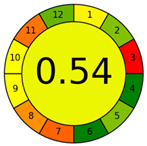

* The missing field in the second pentagram means that the direct method without an extraction process was used. ** National Fire Protection Association (NFPA), lists of codes and standards. *** Penalty points, PP. **** The waste produced by the developed method is collected and sent to a local company for Waste management.

**Table 3:** Greenness assessments of the published methods describing the determination of florfenicol in the urine of animal origin.

Sample	Reference	Treatments and Analytical Conditions	Assessment		
			NEMI	GAPI *	AGREE
calve urine	[1]	V_sample_: 1 mL of diluted urine; internal standard: thiamphenicol; LLE with ethyl acetate; centrifuged; evaporated; dissolved; sonicated; vortexed; HPLC-UV: C-18 (250 × 4.6 mm ID, 5 μm); 223 nm; 1.2 mL/min; acetonitrile/water (40:60, *v*/*v*); 20 µL	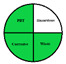	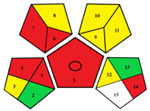	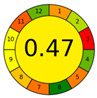
pig urine	[11]	V_sample_: 5 mL; internal standard: enrofloxacin d5; centrifuged; SPE (methanol, water; 0.5 M hydrochloric acid); evaporated; dissolved; vortexed; HPLC-MS/MS: C-18 (150 × 2.0 mm ID, 4 μm); 0.2 mL/min; A (0.1% aqueous formic acid) and B (methanol), gradient elution; 10 µL; run: 30 min	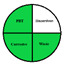	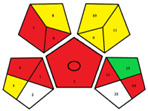	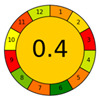
pig urine	[12]	V_sample_: 2 mL; LLE with ethyl acetate; evaporation at 50 °C; purification of 0.1 mL urine extract; TLC; HPLC-UV; silica gel 60 GF_254_ glass plates (10 × 20 cm), 254 nm; C_18_ (250 × 4.6 mm ID, 5 μm), 32 °C; 225 nm; 0.6 mL/min; acetonitrile/phosphate buffer + sodium 1-dodecane-sulfonate + triethylamine (33.3:66.7, *v*/*v*); 50 µL	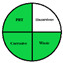	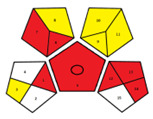	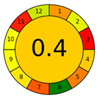
cow, pig and lamb urine	[30]	V_sample_: 5 mL; internal standard: triphenylphosphate; continuous SPE with ethyl acetate; microwave-assisted derivatization with BSTFA and TMCS **; GC-MS; DB-5 (30 m × 0.25 mm ID, 0.25 μm); carrier: helium, 1 mL/min; injection at 280 °C; split mode; run: 30 min	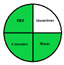	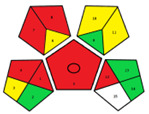	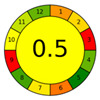
goat urine	[31]	V_sample_: 1 mL diluted urine; internal standard: thiamphenicol; LLE with ethyl acetate; centrifuged; evaporated; dissolved; vortexed; sonicated; HPLC-UV: C-18 (75 × 4.6 mm ID, 3.5 μm) at 20 °C; 224 nm; 1.5 mL/min; acetonitrile/water (18:82, *v*/*v*); 20 µL	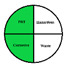	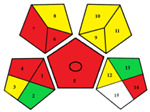	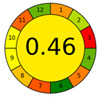
pig and bovine urine	[32]	Synthesis of haptens; purification by gel column chromatography; ID of haptens; coating antigens by active ester method; ID of antigens; preparation and purification of antibodies by precipitation; screening of antibody and coating antigen; centrifugation of urine; LC-MS/MS: HPLC-UV; ultramicro UV spectrometry; 96-well microplates; 450 nm	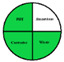	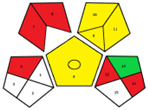	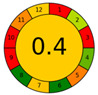

* The blank field means that there was no data to evaluate. ** N,O-Bis(trimethylsilyl)trifluoroacetamide, BSTFA; Trimethylchlorosilane, TMCS.

## Data Availability

Data is contained within the article and Supplementary Material.

## Supplementary Materials

The following supporting information can be downloaded at: https://www.mdpi.com/article/10.3390/ph17040495/s1, Figure S1: Chromatogram of: (**a**) standard solution contending 5.0 μg/mL of florfenicol (FF) and 10.0 μg/mL of chloramphenicol (CAP); (**b**) blank urine sample of pig from the control group; (**c**) pig urine from control group spiked with 5.0 μg/mL of FF and 10.0 μg/mL of CAP; (**d**) urine sample of the treated pig spiked with 10.0 μg/mL of CAP; Table S1: System suitability results for the florfenicol (FF) analytical method using chloramphenicol (CAP) as an internal standard.
