# Supplementary material for: A Green HPLC Approach to Florfenicol Analysis in Pig Urine
Source: Pharmaceuticals (Basel). 2024 Apr 12;17(4):495. doi: 10.3390/ph17040495 (PMC11053663; doi:10.3390/ph17040495)
Supplement: Supplementary file 1 [file pharmaceuticals-17-00495-s001.zip › pharmaceuticals-2935112-supplementary/Figure S1.pdf]

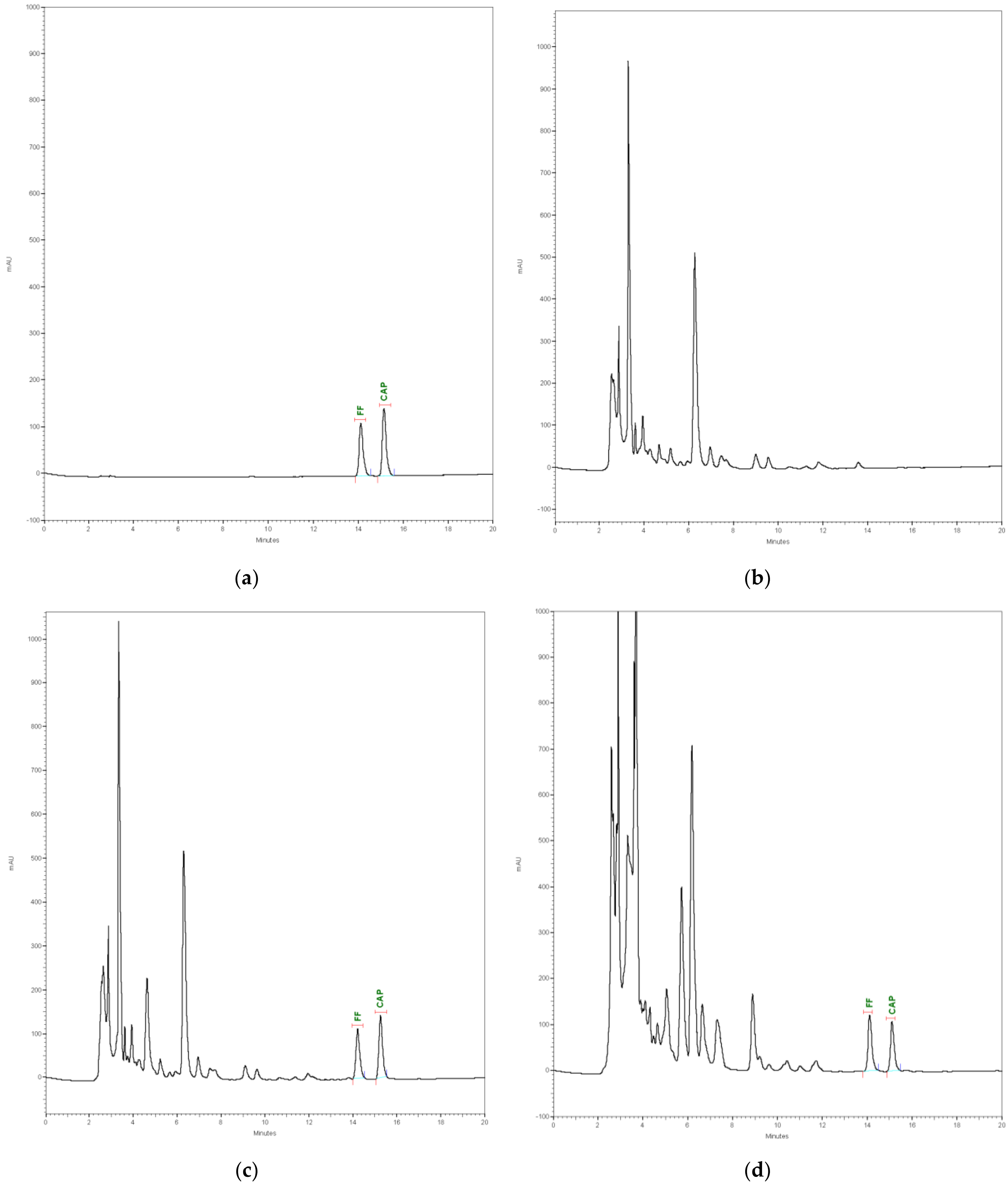

Figure S1. Chromatogram of: (**a**) standard solution contending 5.0 μg/mL of florfenicol (FF) and 10.0 μg/mL of chloramphenicol (CAP); (**b**) blank urine sample of pig from the control group; (**c**) pig urine from control group spiked with 5.0 μg/mL of FF and 10.0 μg/mL of CAP; (**d**) urine sample of the treated pig spiked with 10.0 μg/mL of CAP.
