# Supplementary material for: A Green HPLC Approach to Florfenicol Analysis in Pig Urine
Source: Pharmaceuticals (Basel). 2024 Apr 12;17(4):495. doi: 10.3390/ph17040495 (PMC11053663; doi:10.3390/ph17040495)
Supplement: Supplementary file 1 [file pharmaceuticals-17-00495-s001.zip › pharmaceuticals-2935112-supplementary/Table S1.pdf]

Table S1. System suitability results for the florfenicol (FF) analytical method using chloramphenicol (CAP) as an internal standard.

Parameters *	Reference value	Standard solution		Urine solution	
		average ± SD **	%RSD ***	average ± SD **	%RSD ***
Retention time (t_R)					
FF	-	14.03 ± 0.02	0.13	13.82 ± 0.03	0.20
CAP	-	15.07 ± 0.01	0.09	14.83 ± 0.02	0.15
Resolution (R_S)	≥ 2.0	3.52 ± 0.02	0.71	3.38 ± 0.05	1.35
System repeatability (%RSD)					
FF peak area	≤ 2.0	1374603 ± 5631	0.41	1362532 ± 5750	0.42
CAP peak area	≤ 2.0	1745815 ± 17056	0.98	1696077 ± 19546	1.15
Retention factor (k)					
FF	1-10	6.02 ± 0.01	0.15	5.91 ± 0.01	0.23
CAP	1-10	6.53 ± 0.01	0.11	6.42 ± 0.01	0.17
Symmetry factor (A_s)					
FF	0.8-1.5	1.34 ± 0.01	0.58	1.38 ± 0.01	0.42
CAP	0.8-1.5	1.34 ± 0.005	0.35	1.41 ± 0.01	0.39
Plate number (N)					
FF	> 2000	34829 ± 433	1.24	32385 ± 455	1.40
CAP	> 2000	43686 ± 707	1.62	40080 ± 514	1.28

* System suitability tests were performed in standard (reference) solution and urine (blank sample) solution that contained FF at a concentration of 5.0 μg/mL and CAP at a concentration of 10.0 μg/mL.

** Standard deviation (SD).

*** Relative standard deviation expressed as a percentage (%RSD).
